# Recombinant glucagon: a differential biological activity

**DOI:** 10.1186/s13568-015-0099-2

**Published:** 2015-03-12

**Authors:** Angelina M M Basso, Patrícia B Pelegrini, Fernanda Mulinari, Michelle C Costa, Antonio B Viana, Luciano P Silva, Maria Fatima Grossi-de-Sa

**Affiliations:** Department of Molecular Pathology, University of Brasilia, Brasilia, DF Brazil; Laboratory of Plant-Pest Interaction, Embrapa – Genetic Resources and Biotechnology, Brasília, DF Brazil; Pioneer Union for Social Insertion – UPIS, Planaltina, DF Brazil; Catholic University of Brasilia, Brasilia, DF Brazil; Laboratory of Mass Spectrometry, Embrapa – Genetic Resources and Biotechnology, Brasília, DF Brazil

**Keywords:** Recombinant Glucagon, Peptide expression, Heterologous system, Enterokinase, GST tag, Biological activity

## Abstract

In Brazil, there is a growing demand for specialised pharmaceuticals, and the high cost of their importation results in increasing costs, reaching US$ 1.34 billion in 2012 and US$ 1.61 billion in 2013. Worldwide expenses related to drugs could reach US$ 1.3 trillion in 2018, especially due to new treatments for hepatitis C and cancer. Specialised or high-cost pharmaceutical drugs used for the treatment of viral hepatitis, multiple sclerosis, HIV and diabetes are distributed free of charge by the Brazilian government. The glucagon peptide was included in this group of high-cost biopharmaceuticals in 2008. Although its main application is the treatment of hypoglycaemia in diabetic patients, it can also be used with patients in an alcoholic coma, for those patients with biliary tract pain, and as a bronchodilator. Therefore, in order to reduce biopharmaceutical production costs, the Brazilian government passed laws focusing on the development and increase of a National Pharmaceutical Industrial Centre, including the demand for the national production of glucagon. For that reason and given the importance and high cost of recombinant glucagon, the purpose of this study was to develop methods to improve production, purification and performance of the biological activity of recombinant glucagon. Glucagon was recombined into a plasmid vector containing a Glutathione S-transferase tag, and the peptide was expressed in a heterologous *Escherichia coli* system. After purification procedures and molecular analyses, the biological activity of this recombinant glucagon was examined using *in vivo* assays and showed a highly significant (p < 0.00001) and prolonged effect on glucose levels when compared with the standard glucagon. The experimental procedure described here facilitates the high level production of recombinant glucagon with an extended biological activity.

**Electronic supplementary material:**

The online version of this article (doi:10.1186/s13568-015-0099-2) contains supplementary material, which is available to authorized users.

## Introduction

The development of recombinant proteins has been extremely promising. In 2009, pharmaceutical industries spent US$ 90 billion on the activity evaluation of 400 different drugs. Moreover, in 2012, more than 150 recombinant drugs were approved by FDA (Food and Drug Administration) regulators and/or by the European Medicine Agency (Huang et al. 2012). By 2017, it is expected that over 300 products will be in the biopharmaceutical market, with a value of about US$ 109 billion and an expected growth of US$ 166 billion (Ibarra Cabrera et al. 2013). Worldwide expenses related to drugs could reach US$ 1.3 trillion in 2018, especially due to new treatments for hepatitis C and cancer (Chen, 2014).

According to the World Health Organization, one third of the world’s population has no regular access to essential medicines (Nowbike 2006; Blatt et al. 2012). In Brazil, the Government provides free, high-cost drugs to the population, which are classified into three different categories: basic, strategic and specialised. The specialised components are expensive, which limits access of the population to important medicines. Hence, in 1993, the Program for Specialized Medicaments (SM) became a prospective solution to this problem. However, most of these medicines are imported from other countries, resulting in a large cost to the Brazilian government. Moreover, the expectation is that the amount spent on the importation of SM will continue to grow over the next several years.

In 2009, US$ 884 million was spent on specialised drugs, which is four times the amount (US$ 198 million) spent in 2003 (Valadares 2009). In 2013, US$ 1.61 billion was spent, and in 2014, preliminary data were provided by the Brazilian Ministry of Health showing US$ 1.5 billion for the purchase of SM (Brazil Ministry Health, 2014). Therefore, to reduce high costs associated with imported pharmaceutical products, the Brazilian government released the Technological Innovation Law, which stimulates the development of new biotechnological companies for the production of national biopharmaceuticals (Brazil Ministry Health 2008; de Castro 2014). The Technological Innovation Law is part of a strategic plan to strengthen the pharmaceutical industry, due the increased demand for health public services and consumption (medicines, diagnostics kits, vaccines, etc.). The strategy aimed to replace the imported drugs that were causing a significant impact on the economy and encourage scientific training and public–private partnerships (Victora et al. 2011; Silveira 2012; Reddy 2013; Sundfeld and Souza 2013). In 2012, 34 public–private partnerships were developed to produce several molecules with therapeutic activity, such as anti-retroviral and anti-asthmatic drugs and drugs used to treat rheumatoid arthritis and Crohn and Gaucher diseases, among others (Paiva 2012). In 2013, 63 public-private partnerships were developed, resulting in the production of 63 drugs, vaccines and rapid diagnostic tests, involving 16 public and 41 private laboratories, with an estimated US$ 2.8 billion per year in budget savings (Oliveira 2013). Alongside Brazil, other countries, such as South Korea and India, also used national biosimilar production as an alternative to increase the access of high-cost biopharmaceuticals to the population (IMS 2011) through similar biotechnology. Furthermore, the large accumulation of expired patents related to biopharmaceutical products led to the development of a second generation of drugs: biosimilars (Niederwieser and Schmitz 2010; Utzig et al. 2010). The substitution of classic drugs with biosimilars depends on the criteria established by each country (Rosenberg et al. 2010).

The glucagon peptide could be used in diagnostics tests or for the treatment of alcoholic coma, biliary tract pain, hypoglycaemia (as a bronchodilator), etc. (Guimarães et al. 2011). Furthermore, glucagon has been on the Brazilian SM Program list since 2008. Currently, glucagon is imported from other countries, and initial production of new substances is restricted to universities and research centres (Massi et al. 2013). The main activity of glucagon involves the primary regulation of glucose production in the liver in situations of fasting, exercise and hypoglycaemia (Ramnanan et al. 2011). Hancock and colleagues (2010) indicated that the main glucagon activity was associated with glycemic control in fasting and postprandial situations. Therefore, glucagon utilisation is important since this hormone shows a fundamental role in glycemic control and, consequently, the maintenance of homeostasis.

In this work, the glucagon peptide was expressed and purified from *Escherichia coli* cells using the pGEX vector system. An efficient purification procedure at the laboratory scale for a biologically active Glutathione S-transferase (GST)-glucagon fusion molecule was also performed. The biological activity of the biosimilar molecule was evaluated in *in vivo* tests, showing a prolonged activity when compared with the control (Glucagen NovoNordisk®). This study is the first report in which glucagon is efficiently produced in Brazil using a bacterial system, showing advanced biological activity in *in vivo* assays.

## Materials and methods

### Materials

Glucagon PCR primers were purchased from Integrated DNA Technologies (Coralville, IA, USA) and the FUSION Taq High-Fidelity enzyme was purchased from Finnzymes, Thermo Scientific (Vantaa, Finland). The glucagon expression vector *pGEX4t-3* and the Glutathione Sepharose 4 Fast Flow resin were purchased from GE Healthcare (Inc, Sweden). *E. coli* BL21 (DE3) cells (Invitrogen) were kindly provided by Embrapa Cenargen, Brazil. The expression inductor Isopropyl β-d-1-thiogalactopyranoside (IPTG) was acquired from Invitrogen (Carlsbad, CA., USA). Glucagen®, used as positive control, was obtained from Novo Nordisk A/S (Denmark). Polyclonal anti-glucagon antibodies were purchased from Santa Cruz Biotechnology (Santa Cruz, CA., USA).

#### Cloning of the human glucagon gene

The nucleotide sequence of human glucagon (GenBank GI 30582464), containing 128 bp, was cloned into the synthetic vector *pGEX4t-3* (GE Healthcare). The glucagon coding sequence was then amplified using designed primers containing a *Sal* I site (forward - 5’ GGG GGT CGA CGA TTA CAA AGA TGA TGA TG 3’), a *Not* I site (reverse – 5’ TTT TGC GGC CGC TCA GGT GTT CAT CAG CCA C 3’) and a cleavage site for the FLAG tag. The amplicon was purified, and a recombination reaction was performed to clone the glucagon fragment into the *pGEX4-t3* expression vector (GE Healthcare). The recombinant plasmid was used to transform *E. coli* XLI-Blue cells via electroporation, and transformed cells were initially selected using LB (Luria and Bertani) agar plates [1% (w/v) tryptone, 0.5% (w/v) yeast extract, 0.5% (w/v) sodium chloride, and 1.5% (w/v) agar, supplemented with 100 μg/mL ampicillin, pH 7.0].

#### Recombinant glucagon expression

The construct was inserted into *E. coli* BL21 (DE3) cells through thermal shock. To evaluate the glucagon expression profile, a single colony was inoculated into 10 mL of LB medium containing ampicillin (100 μg/mL) and grown overnight at 37°C (200 rpm). The seed culture was inoculated into 1 L (Litre) of LB medium supplemented with ampicillin, and cells were grown at 30°C during 2 hours. The expression induction was carried out by the addition of IPTG (Isopropyl β-d-1-thiogalactopyranosid; 0.25 mM). After 4 hours of induction, cells were harvested by centrifugation (1680 g for 5 min at 25°C). The supernatant was discarded, and the pellet was resuspended in 1X sample buffer [10% glycerol (w/v), 2-10% mercaptoethanol (v/v), 2.3% sodium dodecyl sulfate (w/v), 124 mM Tris-Base, 0.01% bromophenol blue (w/v)] and boiled for 5 min. After that, soluble and insoluble proteins were separated by centrifugation (13171 g for 5 min), and protein molecular masses were analysed by Coomassie-blue stained, 12.5% acrylamide SDS-PAGE gels (Sambrook and Russell 2006). Glucagon expression levels were determined by densitometry using the ImageScannerIII (GE Healthcare Inc., Sweden) and ImageMaster2D Plattinum 7.02 and LabScan 6.0 (GE Healthcare Inc., Sweden) software for data analyses.

Bioreactor glucagon expression was performed. Larger lab-scale peptide production was obtained using the Wave Bioreactor™ 20/50 EHT system (GE Healthcare, Brazil). A series of parameters were adjusted to optimise glucagon expression in the Wave Bioreactor, such as the swing angle (30°), speed (30 rpm), temperature (30°C) and airflow (0.1 ppm for 30 seconds every 1 hour). Peptide expression was induced with the addition of IPTG (0.25 mM), and cells were harvested after 4 hours.

#### Glucagon purification

Recombinant glucagon was purified by Glutathione Sepharose 4 Fast Flow (GE Healthcare Inc., Sweden) affinity chromatography according to the manufacturer’s instructions. Purified samples were dialysed overnight in a tube with a cut off of 12000–14000 kDa (Cheshire Sciences, UK), lyophilised and stored at −20°C for quantification. Each sample concentration was previously estimated using Qubit methodology (Invitrogen, USA). Quantified samples were used for the digestion procedure with enterokinase.

#### Western blot assays

Purified GST-FLAG-Glucagon and non-purified protein concentrations were estimated using the Bradford method (1976). Protein molecular mass was estimated as previously described. Glucagon was detected using the polyclonal anti-glucagon antibody (Santa Cruz, CA., USA) and alkaline phosphatase-conjugated anti-goat IgG (GE Healthcare Inc., Sweden). The Alkaline Phosphatase Conjugate Substrate Kit (BioRad, CA., USA) was used according to the manufacturer’s instructions.

#### Cleavage of the fusion protein

The cleavage reaction for the fusion protein was performed using the enzyme enterokinase EKMax (Invitrogen, USA) at a concentration of 1 U per 20 μg of purified recombinant peptide and incubated at 22°C overnight. A 7-16% Tris-Tricine gel (Schangger 2006) was used to analyse the cleavage reaction.

#### Protein sequencing

After cleavage, the GST-FLAG and Glucagon proteins were diluted in 0.1% trifluoroacetic acid and purified by reverse-phase ultra fast high performance liquid chromatography (RP-UFLC) (RP-UFLC LC-20 AD/T LPGE kit, Shimadzu Corporation Kyoto, Japan) using a Vydac C-_18_TP column. Retained proteins were eluted with a linear gradient of acetonitrile (0-100%). Samples were then subjected to speed-vac concentration, and major fractions were mixed with a saturated matrix solution of alpha-cyano-4-hydroxycinnamic acid (1:3) and spotted (0.5 μl) onto an MTP AnchoChip var/384 matrix-assisted laser desorption ionisation (MALDI) target plate. The monoisotopic molecular mass of glucagon was determined by MALDI-time of flight TOF/MS tandem mass spectrometry (MALDI-TOF/MS) using an AutoFlex Speed (Bruker Daltonics, Germany) controlled by the FlexControl 3.0 software. The MS spectra were acquired in the positive ion reflector mode at a laser frequency of 100 Hz, and the MS/MS spectra were obtained by LIFT^TM^ mode, both with external calibration using the peptide calibration standard I (Bruker Daltonics, Germany). Data were analysed using the Flex-Analysis 3.0 software.

#### *In vivo* bioassays

*In vivo* bioassays were performed following the methodology described by Hinke et al. (2000) with modifications. Briefly, recombinant glucagon (7.1 nmol/kg), commercial glucagon (positive control) and dH_2_O (negative control) were injected into three groups of five male Wistar rats, respectively. After administration, glucose levels were monitored at 0, 10, 20 and 40 minutes. The differences in glucose levels (differences between 10, 20 or 40 minutes and the baseline time) over time were calculated and evaluated. When the recombinant glucagon was used, the blood glucose level was maintained at a 1.3-fold increased level 40 minutes after injection, whereas it decreased to similar levels as the negative control after 40 minutes when the commercial glucagon was used. The biological test was repeated three times.

In this study, to biological assay the initial samples contained recombinant glucagon with a FLAG-GST tag. The fusion between FLAG-GST and recombinant glucagon was removed from digestion with enterokinase. After this step, samples were evaluated for *in vivo* biological activity. The FLAG-GST tag and recombinant glucagon were in the same sample to *in vivo* biological activity, but not were fusioned.

The study was previously approved by the Pioneer Union for Social Insertion – UPIS Committee for Ethical Use of Animals (CEUA), and the proposal (#071-11) entitled “Biological activity evaluation of recombinant peptide glucagon” was approved on August 12, 2011. Therefore, the reported experimental procedures comply with ethical procedures for animal use in *in vivo* experiments (Ellery 1985).

Statistical analyses were based on the generalised linear mixed model test (R Development Core Team 2010) and were used to validate the differences between values obtained by the different groups. In addition, analyses were performed to assign a fold-difference value comparing the increased glycemic levels seen at 10, 20 and 40 minutes with the basal levels, considering the random variation of rats. P < 0.05 was considered statistically significant. R statistical packages were used to validate the data.

## Results

### Glucagon expression and purification

**Figure 1:**
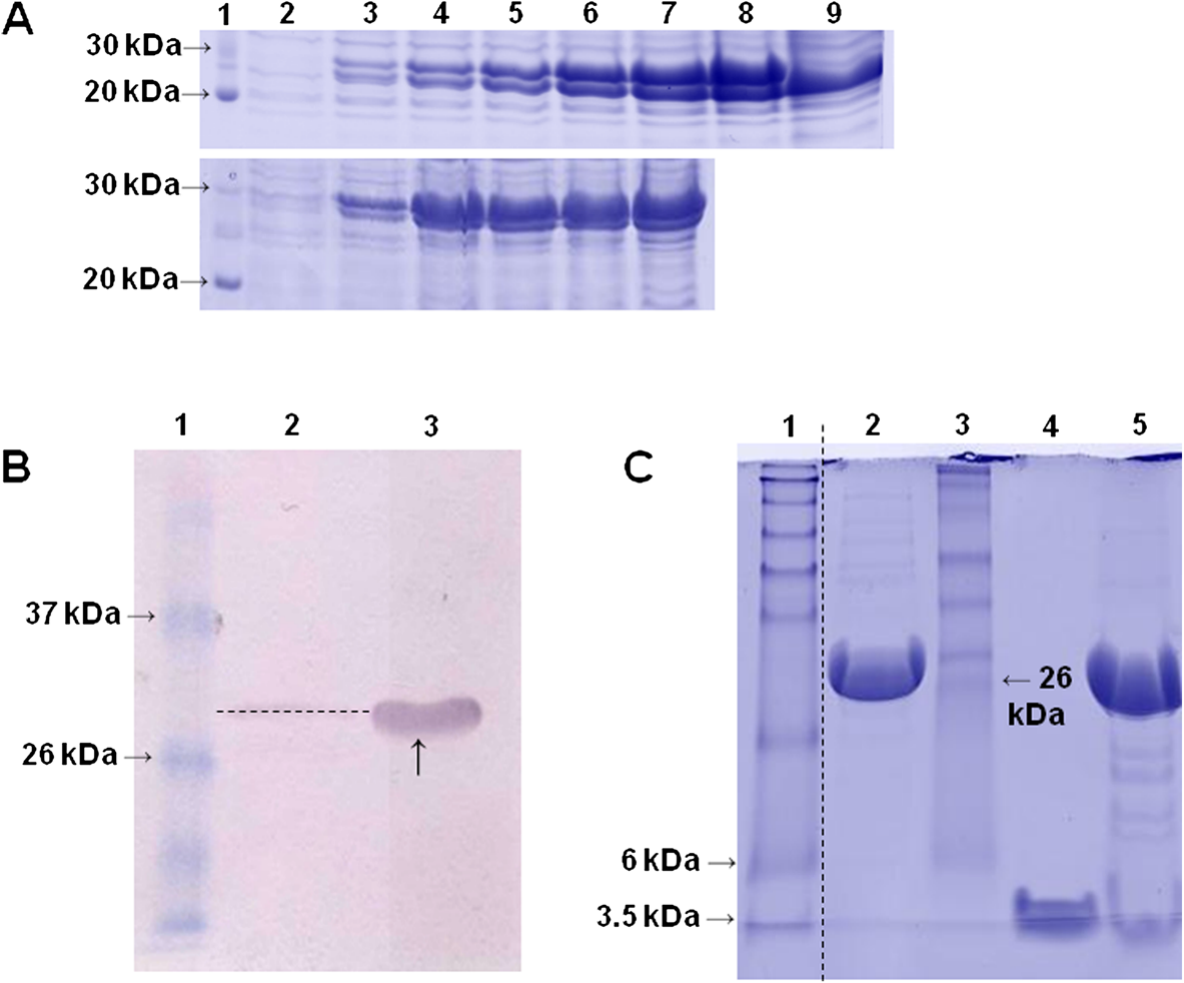
**Recombinant glucagon peptide expression profiling and analysis. (A)** Glucagon expression after different incubation times. Upper lane: protein expression using 1 L of culture media; Lower lane: protein expression in the bioreactor (5 L). Lane 1: BenchMark Protein Ladder (Invitrogen); Lanes 2 to 9 indicate expression protein patterns after 0 h, 1 h, 2 h, 3 h, 4 h, 5 h, 6 h and overnight incubation, respectively. **(B)** Western blotting of glucagon using anti-glucagon antibodies. Lane 1: Pre-Stained Protein Ladder (Invitrogen); Lane 2: purified GST protein (3 μg); Lane 3 purified non-digested glucagon fused with the GST protein (3 μg) arrows indicate recombinant glucagon fused with GST protein. **(C)** Glucagon digestion to separate the peptide from the GST tag in Tris-Tricine gel. Lane 1: Seeblue Plus2 Pre Stained Standard (Invitrogen); Lane 2: purified non-digested glucagon fused with the GST protein (5 μg); Lane 3: Pre-Stained Protein Ladder (Invitrogen); Lane 4: commercial glucagon (Glucagen, NovoNordisk, 5 μg); Lane 5: digested glucagon (40 μg total digestion).

After expression, it was observed that 40 to 50% of total soluble product corresponded to the glucagon peptide, which, including the GST fusion protein, presented a molecular mass of 31.44 kDa (Figure 1A; Additional file 1: Figure S1). After purification using affinity chromatography, densitometry analyses indicated that the GST-glucagon corresponded to 90% of the purified product. Western blotting assays using both anti-GST and anti-glucagon antibodies confirmed the presence of the GST-fused glucagon (Figure 1B). Later, the cleavage of glucagon from the GST fusion protein using the enterokinase enzyme produced a 3.5 kDa-peptide, as revealed by a 16% Tris-tricine gel (Figure 1C).

### Amino acid sequencing of glucagon

After hydrolysis, the glucagon peptide was purified using UFLC (ultra-high performance liquid chromatography), and fractions were collected. The ion corresponding to the peptide was present in a fraction that eluted at 26 minutes of separation, and it was further detected by MALDI-TOF (Matrix Assisted Laser Desorption Ionisation Time-of-Flight) MS (Additional file 2: Figure S2). Hence, 16 amino acid residues from the N-terminus of glucagon (H-S-Q-G-T-F-T-S-d-Y-S-K-Y-l-d-S) were determined by *de novo* sequencing, indicating that the predicted digestion was performed as expected.

### *In vivo* bioassays

**Figure 2:**
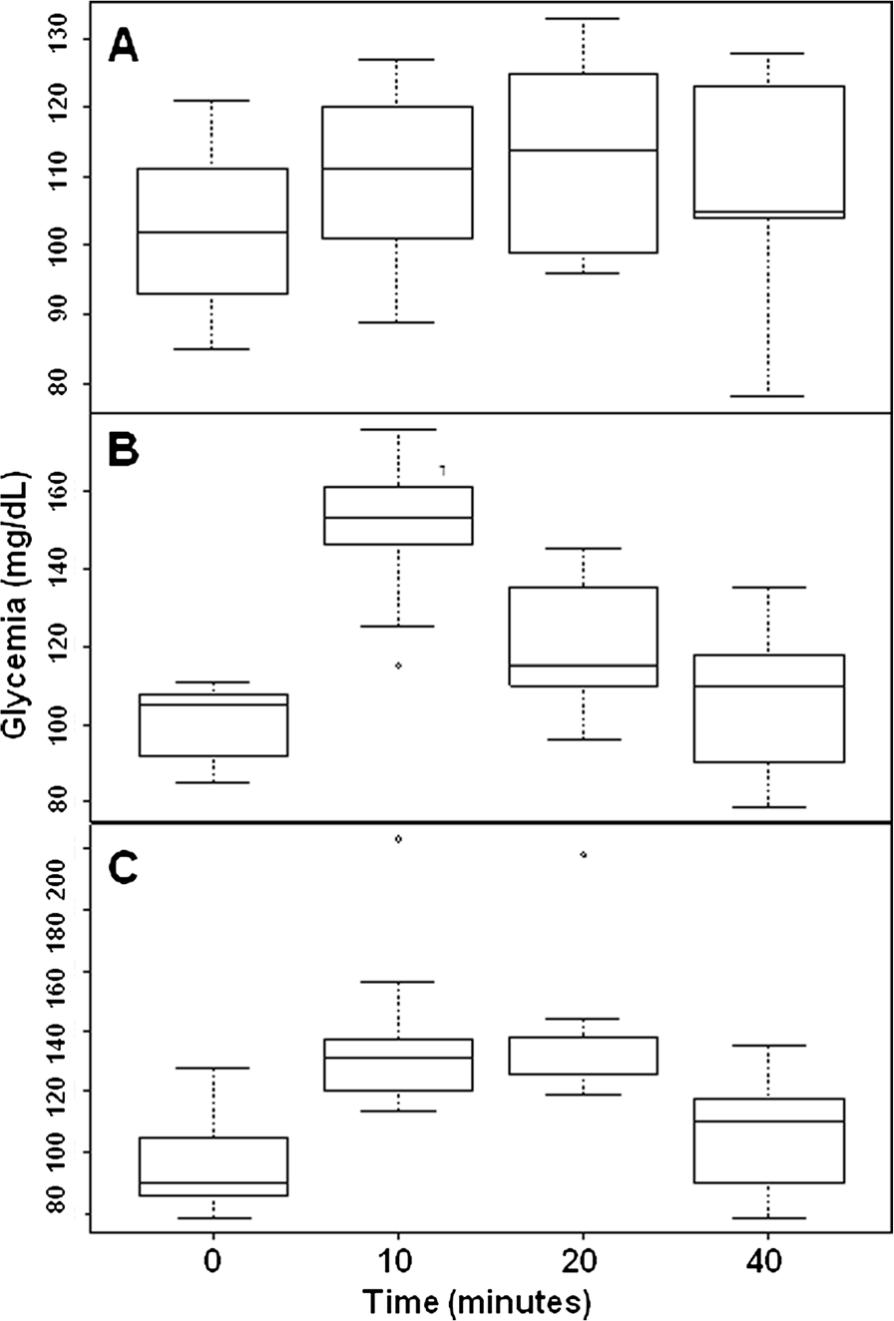
**Statistical model analysis of the biological activity test based on glucose levels during specific times after of samples administration. (A)** Negative control. **(B)** Positive control. **(C)** Recombinant glucagon. p > 0,00001.

GST-Glucagon activity was evaluated by measuring glycemic levels in rats before and after exposure to the glucagon molecule. Both the recombinant peptide and the commercially available glucagon (NovoNordisk®) induced a similar blood glucose peak right after injection. Nevertheless, the recombinant glucagon maintained blood glucose levels at a 1.3-fold increased level 40 minutes after injection, whereas commercial glucagon decreased to the same levels of negative control after 40 minutes (Additional file 3: Figure S3). Statistical analyses demonstrated that the differential time profile of glucose levels between the recombinant glucagon and the commercial glucagon was significant (Figure 2A-C).

### Glucagon expression using the WAVE Bioreactor

To evaluate the expression level of recombinant glucagon at a higher scale with controlled conditions, the WAVE bioreactor (GE Healthcare) was used at a scale up of 5 L (Figure 1A, lower lane). After expression and purification, the final yield of recombinant glucagon in the bioreactor reached 7 mg/L.

## Discussion

Among the seven emerging countries in the biopharmaceutical market (Russia, China, Turkey, Mexico, South Korea, India and Brazil), a growth from US$ 5.3 billion in 2010 to US$ 13.6 billion in 2015 is estimated (International Market Analysis Research & Consulting, 2010). Biosimilar sales are expected to reach between US$ 1.9 - 2.6 billion (IMS 2011) in 2015. In 2016, the medicines market will spend US$ 1.2 trillion, most of which are expenses focused on cancer, diabetes and asthma drugs (IMS Institute 2012).

The cost of drugs used for diabetes treatment (insulin, glucagon, and others) reached US$ 548 billion in 2013. In the United States, it is estimated that the diabetes drug market will expand by about US$ 125 million (Biodel 2013; Hassan et al. 2014). Statistical data suggest a worldwide growth from 382 million diabetic people in 2013 to 592 billion people in 2035 (Ledford 2013; Guariguata et al. 2014). There is an estimated 200.000 people/year that are hospitalised due to severe hypoglycaemia, and only 20% of this number use the glucagon emergency kit. One of the main causes for the lack of access to the kit is the elevated price (Biodel 2013). The first glucagon recombinant regimen was created by the NovoNordisk® Company in 1989 using a yeast heterologous system (*Saccharomyces cerevisiae*) (patent n°4.826.763). In 1994, the Eli Lilly® Company used an *E. coli* heterologous system to produce recombinant glucagon (patent n°5.512.549). Today, recombinant glucagon is expressed in many countries for high scale production purposes, mainly own production (Agarwal et al. 2014) and such that is reported by When and colleagues (2001).

The glucagon production reached 40 to 50% of the total protein expression, with concentrations slightly above the 39% reported by Ohana and colleagues (2011), which evaluated different protein expression yields with differential tag systems. The tac promoter (de Boer et al. 1983) used in the expression vector contributed to the high level of glucagon production. It is already known that the tac promoter is strong and one of the most widely used in research and industry (Huang et al. 2012).

Bioreactors have been used in the recombinant peptide industry for more than a decade, mainly due to the flexibility of use reducing costs, labour and contaminations. However, the major limitations are the amount of oxygen used during expression and the low refrigeration capacity (Dreher et al. 2014). An initial peptide expression in bioreactors was also performed using up to 5 L of culture media. The ultimate goal of establishing a methodology for producing biopharmaceuticals is scale-up. Hence, the expression yielded 122 mg of semi-purified peptide, a 3.4-fold increase compared with the expected value observed earlier for the 1 L expression. This result suggested that the expression of glucagon in bioreactors using bacterial cells might provide better conditions for peptide production, indicating that the methodology used can easily be applied at industrial levels.

It is estimated that 77% of *E. coli* expression proteins are insoluble (Cuozzo and Soutter 2014). However, some studies showed high levels of soluble peptide production when fused with tag proteins, such as Thioredoxin (Trx) and GST (Gao et al. 2010). Hence, the glucagon peptide was expressed in the soluble phase partly due to the addition of a GST tag. At the end of the process, achieving satisfactory amounts of protein does not mean success (Rosano and Ceccarelli 2014). It is necessary to evaluate the biological activity to validate the process (Nausch et al. 2013). Here, we demonstrate that the recombinant GST-glucagon displayed high glycemic levels for a longer period of time than the commercial glucagon. For a biosimilar, it was expected that recombinant glucagon here produced and the positive control (commercial glucagon) should demonstrate similar glucose levels during the evaluated time, which was not observed in this study.

The analysis of recombinant proteins fused with GST in biological assays has been earlier described, with no interference of the targeted protein's activity. Recently, two antitumoral proteins, granzyme B (GrB) and perforin (PFP), earlier known by their ability to inhibit cell viability, were cloned into the *pGEX-4 T-1* vector and expressed in bacterial systems. The fusion proteins GST-GrB and GST-PFP were later challenged against Hep-2 cells, showing significant decreases in laryngeal cancer cell growth (Li et al. 2014). Using GST as a control, the assay also demonstrated the inability of the fusion protein to interfere with recombinant protein activities.

Therefore, the application of bacterial systems for the expression of human peptides fused with GST proteins is becoming a promising strategy in the production of recombinant molecules with potential for biopharmaceutical products. A previous report showed that a recombinant glucagon-like peptide-1 analogue (KGLP-1), also used on diabetes therapy, was expressed in *E. coli* cells as a fusion protein with GST (Liu et al. 2014). In this study, digestion reactions were performed to remove GST from glucagon and confirmed by Tris-Tricine gel (Figure 1C). Furthermore, in biological activity, the recombinant glucagon in the presence of GST tag, but not fused to it, showed prolonged high glycemic levels when compared with the positive control (Glucagen® NovoNordisk). The removal of tags from recombinant proteins is also critical, as it may interfere with the shape and activity of the protein of interest. Moreover, recombinant proteins with permanent tags are not allowed by the standards of therapeutic proteins (Walls et al. 2011; Einsfrldt et al. 2011). Therefore, the GST tag was removed from the fusion with the peptide glucagon using enterokinase. According to Hosfield and Lu (1999), enterokinase presents a cleavage efficiency, when the first amino acid residue of a protein fused with flag-tag is histidine, which is the case of recombinant glucagon, the cleavage efficiency of enterokinase is 74%. Moreover, according to Kahn (2009), of the 29 amino residues, the first amino acids close to the N-terminal portion are essential for biological activity, mainly histidine (position 1), asparagine (position 9) and serine (position 16). If glucagon was fused to GST, the histidine amino acid could not be connected to the glucagon receptor, and the biological activity would be partially impaired, which was not observed here.

All patents of technologies used in this work (Table 1) were deposited in 1997. Thereby, the patents expire in 2017, allowing the use of this protocol to obtain recombinant glucagon. The estimated value spent (Table 2, Additional file 4: Table S1) per mg of recombinant glucagon in this work was US$ 175.28. However, this value can be reduced to US$ 22.10 (see Table 2). In Brazil, the commercial value of recombinant glucagon is US$ 56.60, 2.5 times more expensive than recombinant glucagon. This value can be changed because the recombinant glucagon was semi-purified and total purification can increase the value.

**Table 1:** **Technologies used in the process to obtain recombinant glucagon and respective patents**

**Technology**	**Patent number**	**Patent year**	**Patent expiration**
pGEX4t-3 t vector	US 5654176	1997	2017
GST Tag	US 5654176	1997	2017
FLAG Tag	US 4703004	1987	2007
BL21(DE3) strain	US 5693489	1997	2017
Enterokinase Enzyme	US 5665566	1997	2017
Glutathione Sepharore Resin	US 5654176	1997	2017
Glucagen®	US 5652216	1997*	2017

*The first patent filed was in 1989. This is an *evergreening* patent, a type of patent with novel technology is added to previous one. Basso (2012)

**Table 2:** **Products used for recombinant glucagon expression and respective costs for 1 litre expression**

**Product**	**Price (US$)**	**Amount used**	**Final price spent (US$)**
Ampicillin	134.33	100 mg	2.68
Benzamidine	207.92	180.225 mg	7.49
Enterokinase 1	781.50	1U/20 μg fusioned protein	1094.11*
Enterokinase 2	781.50	1U/1 mg fusion protein	21.87*
Yeast extract	56.60	5 g	1.14
Reduced glutathione	6135.00	3.07 g	14.21
IPTG	86.03	59.57 mg	5.12
KCl	47.54	3 mg	0.0002
KH_2_PO_4_	107.54	3.67 mg	0.007
NaCl	47.54	5.12 g	0.97
Na_2_HPO_4_	125.29	21.29 mg	0.005
PMSF	181.13	261.29 mg	9.46
Tips	16.98	15 units	0.25
Glutathione Sepharose	1864.90	4 mL	74.59
Tryptone	128.31	10 g	5.14
TrisHCl	93.59	6.05 g	2.26
Water, equipment and others			9.53
**Total with enterokinase 1**			1226.96 (**/7 = 175.28)
**Total with enterokinase 2**			157.72 (**/7 = 20.10)

Prices obtained from the companies Invitrogen, GE and Sigma. *Value may change depending on the concentration used for digestion. In this work, we used manufacturer’s instructions (1U/20 μg), but literature reports (Ehrhardt et al. 2000; SUN et al. 2011) indicate 1U/mg recombinant protein to digestion. Thereby, this concentration was used to calculate this value in this condition. **Total value divided by 7, the recombinant protein total obtained in the 1 litre expression (7 mg in 1 litre) to indicate the value for 1 mg. Basso (2012)

In this work, the glucagon peptide was expressed in a soluble form using a pGEX vector in a bacterial system, yielding a high concentration of the purified product. Moreover, the recombinant glucagon showed a differentiated activity on glycemic levels when compared with the positive control, in the presence but not fused to the GST-tag. Further studies aimed to better understand the cause of this extended effect on glycemic levels and the role of GST are being performed.

Many recombinant biopharmaceuticals have been developed for use in biotechnology. However, after the expiration of several pharmaceutical patents, a variety of biosimilars have been produced. Some strategies are emerging for protein production at a large-scale to obtain higher levels of expression. Hence, in this report, we describe, for the first time, the production of the biosimilar glucagon hormone in a heterologous system by a Brazilian research group. Moreover, an improved expression and purification of the peptide is reported, which could support, in the future, the treatment of patients with several different diseases and will decrease Brazilian importation costs with medicaments. Furthermore, local production of the biosimilar glucagon will stimulate the development of national pharmaceutical industries. The peptide in the presence of GST, but not fused, showed a prolonged activity when compared with the positive control. This differential activity may be beneficial because it reduces the discomfort caused by the increase and decrease of glucose levels after commercial drug administration. However, further studies will indicate potential adverse effects of recombinant glucagon.

## Additional files

Additional file 1: Figure S1. Recombinant glucagon peptide expression profiling. Soluble and insoluble expression of the glucagon samples. Lanes 1 and 6: Prestained SDS-PAGE Standards Low Range (Bio-Rad); Lane 2: Expression of the soluble glucagon; Lane 3: soluble fraction of expressed proteins in a non-induced sample; Lane 4: insoluble fraction of expressed glucagon; Lane 5: insoluble fraction of expressed proteins in a non-induced sample; Arrows indicate recombinant glucagon fused with the GST protein. Additional file 2: Figure S2. Mass spectrometry sequencing using MALDI-TOF/TOF (LIFT^TM^ mode). Sequencing part of the fraction of the 1991.83 Da of the glucagon peptide showing the N-terminal portion (y) starting from amino acid H(y1) and reverse sequencing (b) starting from amino acid Q (b2) (despite the absence of b1, the same reveals its presence in y series). The calibrant used the peptide calibration standard II Mixture (Bruker Daltonics), detection range m/z 1000–4000. The analysed fraction retention time was 26 min. Additional file 3: Figure S3. Analysis of glycemic levels using the *in vivo* biological activity test. This assay used four times (0, 10, 20 and 40 minutes after sample administration). Positive control (Glucagen® NovoNordisk), Negative control (dH_2_O). Additional file 4: Table S1. Apparatus used for recombinant glucagon and costs for 1litre expression.
