# Supplementary figures and images for: Recombinant glucagon: a differential biological activity
Source: AMB Express. 2015 Mar 12;5:20. doi: 10.1186/s13568-015-0099-2 (PMC4385203; doi:10.1186/s13568-015-0099-2)

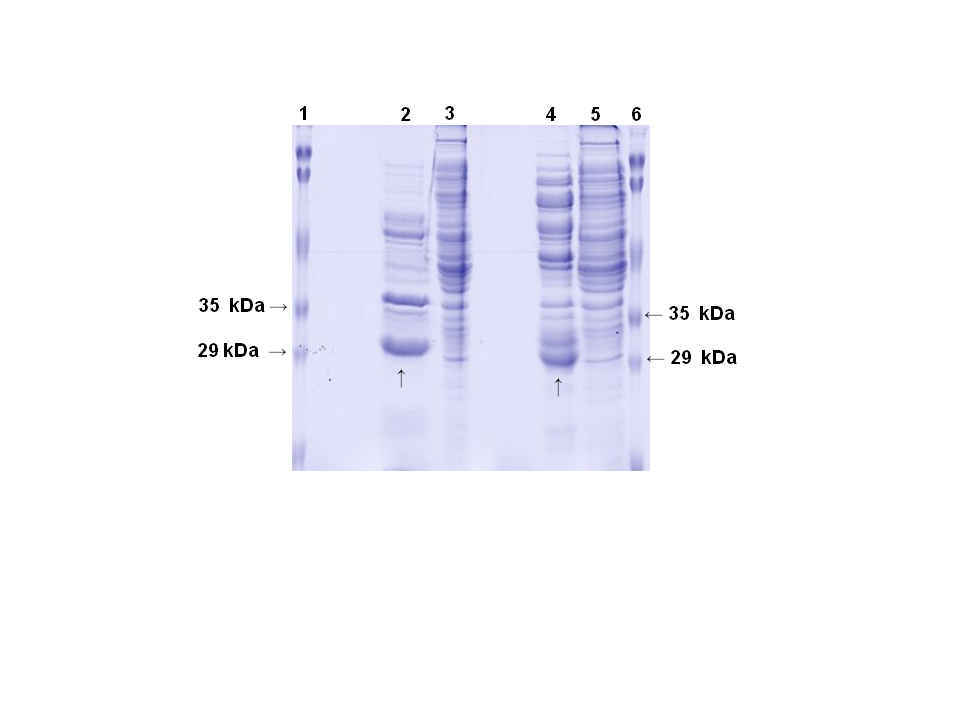

Supplement: Additional file 1: Figure S1. — Recombinant glucagon peptide expression profiling. Soluble and insoluble expression of the glucagon samples. Lanes 1 and 6: Prestained SDS-PAGE Standards Low Range (Bio-Rad); Lane 2: Expression of the soluble glucagon; Lane 3: soluble fraction of expressed proteins in a non-induced sample; Lane 4: insoluble fraction of expressed glucagon; Lane 5: insoluble fraction of expressed proteins in a non-induced sample; Arrows indicate recombinant glucagon fused with the GST protein. [file 13568_2015_99_MOESM1_ESM.tiff]

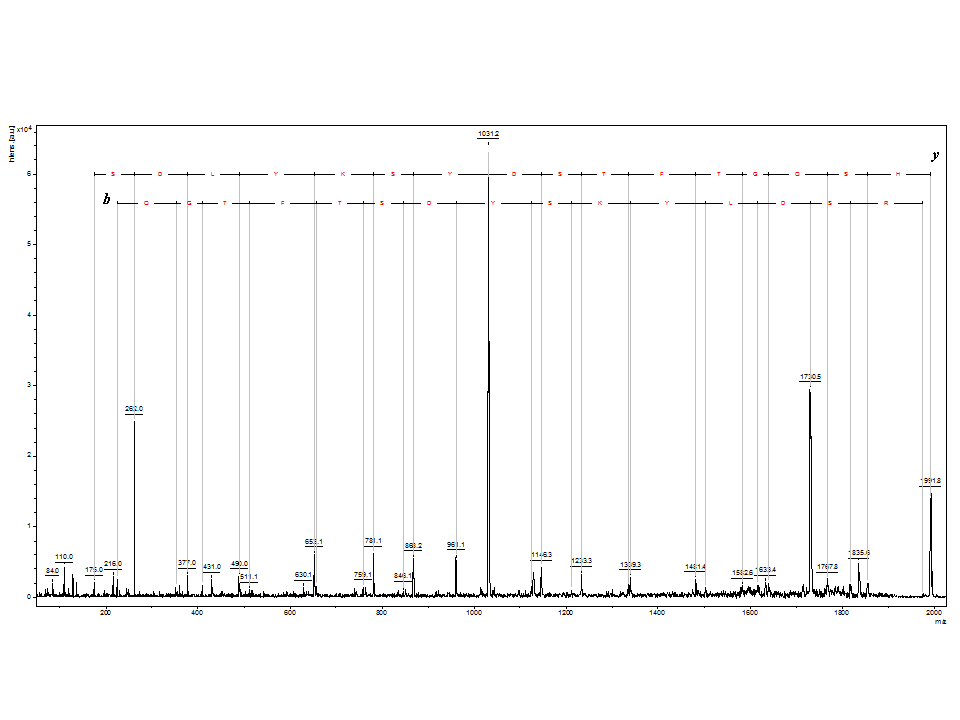

Supplement: Additional file 2: Figure S2. — Mass spectrometry sequencing using MALDI-TOF/TOF (LIFTTM mode). Sequencing part of the fraction of the 1991.83 Da of the glucagon peptide showing the N-terminal portion (y) starting from amino acid H(y1) and reverse sequencing (b) starting from amino acid Q (b2) (despite the absence of b1, the same reveals its presence in y series). The calibrant used the peptide calibration standard II Mixture (Bruker Daltonics), detection range m/z 1000–4000. The analysed fraction retention time was 26 min. [file 13568_2015_99_MOESM2_ESM.tiff]

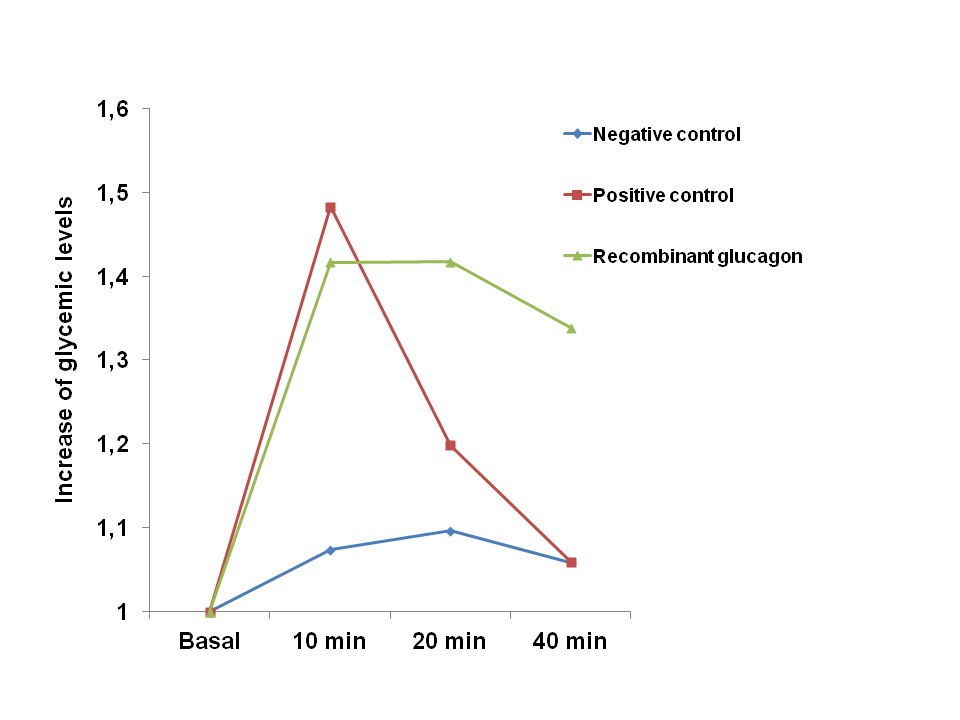

Supplement: Additional file 3: Figure S3. — Analysis of glycemic levels using the in vivo biological activity test. This assay used four times (0, 10, 20 and 40 minutes after sample administration). Positive control (Glucagen® NovoNordisk), Negative control (dH2O). [file 13568_2015_99_MOESM3_ESM.tiff]

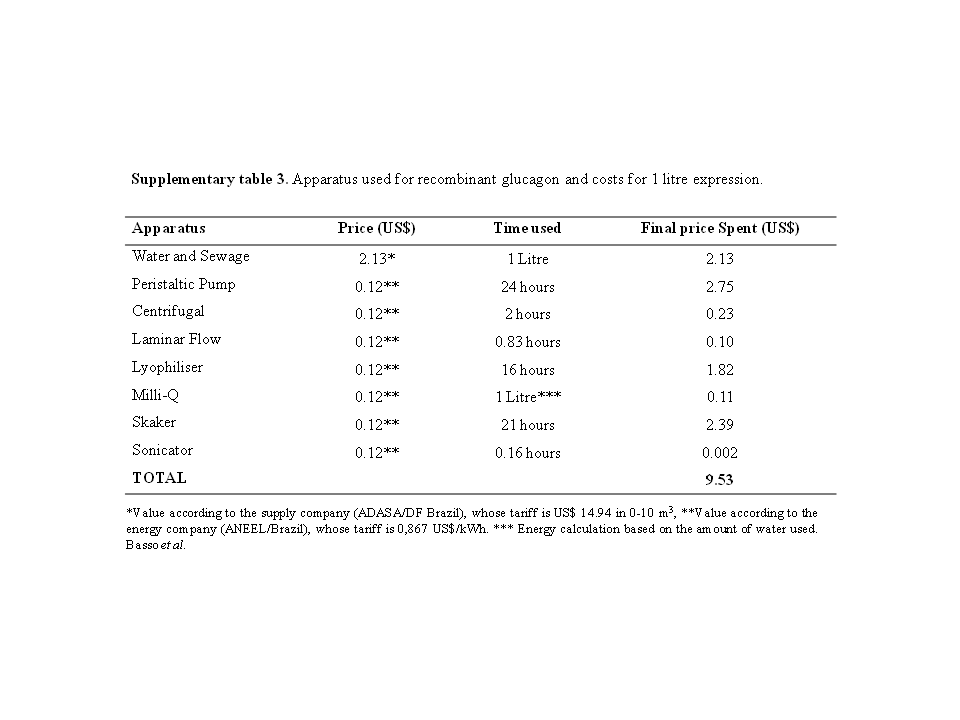

Supplement: Additional file 4: Table S1. — Apparatus used for recombinant glucagon and costs for 1litre expression. [file 13568_2015_99_MOESM4_ESM.tiff]
